# Commentary: A Compositional Neural Architecture for Language

**DOI:** 10.3389/fpsyg.2020.02101

**Published:** 2020-09-02

**Authors:** Elliot Murphy

**Affiliations:** ^1^Vivian L. Smith Department of Neurosurgery, McGovern Medical School, University of Texas Health Science Center, Houston, TX, United States; ^2^Texas Institute for Restorative Neurotechnologies, University of Texas Health Science Center, Houston, TX, United States

**Keywords:** gain modulation, neural oscillations, semantics, syntax, compositionality

Martin (2020) defends a model of linguistic computation relying on gain modulation. I will argue that any gain modulation model of language faces a number of internal conceptual and empirical difficulties, and that our current understanding of gain modulation does not support its use as a core component of linguistic computation.

## The Proposal

Martin (2020) defends a multidimensional coordinate system for language based on neurophysiological models of sensory processing. With ambitious scope, Martin aims to connect theories of processing and neural implementation, an extraordinarily challenging undertaking. Martin maintains that neural trajectories (of these coordinates) encode *sensory, motor*, and *abstract linguistic states*. Gain modulation tunes the path of these trajectories in accordance with behavior. Martin claims that increasingly abstract linguistic structures (from syllables to morphemes to words) are inferred via gain modulation which she defines as “the way neurons combine information from two or more sources.”

Martin (2020) reports how gain modulation underlies “coordinate transform between sensory modalities and between sensory and motor systems.” She cites classic work on cellular dynamics (Salinas and Abbott, 2001), but does not cite other work (e.g., Dayan and Abbott, 2001) where the limitations of gain modulation are stressed, e.g., gaze-dependent gain modulation of retinotopic visual receptive fields is well-reported, but the relevance of gain modulation to higher cognition is not established [(Dayan and Abbott, 2001), p. 17].

Martin (2020) makes an additional claim that “gain modulation also offers a built-in system for predictive coding,” citing Friston (2005); more recent, updated accounts (e.g., Friston, 2018) rely on an interplay of a range of other processes. Moreover, Friston's ventures into language (i.e., Friston et al., 2017) rely on other neural mechanisms (belief propagation in neuronal process theories, phase precession, theta-gamma coupling) employing distinct softmax functions, logarithmic transforms, and linear algebra (implemented via firing rate functions, non-linear postsynaptic responses, and neuronal connectivity, respectively).

Potential advantages of Martin's model are the multiple-realizability of gain modulation and the fact that her model relies on simple changes to a highly conserved, generic neural process—an elegant framework for language evolution.

## A Reassessment

Ferguson and Cardin (2020) provide a comprehensive review of gain modulation, pointing to a common set of mechanisms: GABAergic inhibition, synaptically driven fluctuations in membrane potential, and changes in cellular conductance. Ferguson and Cardin (2020) note that diverse cortical functions such as information integration across cognitive, sensory, and motor systems seem to be performed through gain modulation. Further, regulation of neural gain can provide “an integration mechanism whereby information from multiple sources can be non-linearly combined via multiplicative modulation of the cell's response to inputs” [Ferguson and Cardin, 2020, p. 81]. Martin (2020) defends an additive, rather than a tensor-based multiplicative, formulation of linguistic compositionality. Ferguson and Cardin (2020) review how visual properties that must be *decoded separately* in higher-order areas of non-human primate visual cortex are combined multiplicatively (e.g., object identity), whereas parts that must be *integrated* (e.g., object sub-parts) are combined additively. It may be that additive-only models can capture integrative language functions, but that structural (hence, syntactic) separation may require multiplicative modulation.

Ferguson and Cardin (2020, p. 88) note that gain modulation may be able to *increase information transmission* and provide *computational efficiency* within a given network. Nevertheless, it remains unknown how gain modulation at the single-cell level contributes to population coding that can be read out by downstream targets. The authors conclude that “gain is regulated by a wide range of influences, including attention, learning, locomotion” [(Ferguson and Cardin, 2020), p. 89]. While the role of gain modulation in sensory-to-motor conversions is well-established (evidence that left premotor regions are implicated in language comprehension can be found in Keitel et al., 2018; see also Molinaro and Lizarazu, 2018; Woolnough et al., 2019; Poeppel and Assaneo, 2020), and information transfer between motor and higher-order cortical structures has been shown [crucially via cross-frequency coupling; (Keitel et al., 2018)], gain modulation has not been implicated in higher-order aspects of language in non-motor regions (i.e., components of the cortical language network). As such, it seems premature to use gain modulation as a core component of higher-level linguistic computation.

Ferguson and Cardin (2020, p. 89) also note that “the precise relationship between gain modulation of single neurons and the encoding and transmission of information at the population level is not well-understood,” and that “the reliability and repeatability of gain modulation of single neurons and cortical networks is unknown.” Given this, it seems not well-motivated to stipulate a close connection between gain modulation and oscillatory activity in language. Since gain modulation appears relevant to enabling networks of neurons to produce distributed representations of stimulus features (as opposed to generating inferences to higher abstract structures from such features), it may be that gain modulation goes the way of entrainment: Initially viewed as a crucial mechanism for all manner of linguistic processes (from syllables to sentences) before being relegated by some to a role in (possibly higher-order) speech perception (Keitel et al., 2018; Meyer et al., 2019; Murphy, 2020).

Advancing on and moving away from previous theoretical assumptions (Martin, 2016), Martin (2020) now proposes that cortical oscillations structure speech input into linguistic representations via gain-modulated multiplexing. Multiplexing models of language have been proposed elsewhere, and it is unclear how (Martin, 2020) *gain-modulated multiplexing* is a step forward. For instance, a range of papers (Murphy, 2015, 2016, 2018, 2020; Murphy and Benítez-Burraco, 2017; Benítez-Burraco and Murphy, 2019) utilize multiplexing as operationalized via cross-frequency coupling, and there is ample empirical support for the involvement of this mechanism in aspects of language processing beyond semantic compositionality, such as prediction and syntactic categorization (see Murphy, 2020 and references therein). In addition, testing the claims in Martin (2020) will require a deeper understanding of the relationship between gain modulation and processes that we can more easily record in the human brain. Lastly, while Martin (2020) presents a list of non-specific predictions pertaining to “low frequency power” increases during “structure” building (an eventuality which would not exclusively support a gain modulation model), Murphy (2020) presents specific predictions about distinct low frequency band increases, suppressions, and coupling dynamics, and can hence be evaluated more clearly.

## Author Contributions

The author confirms being the sole contributor of this work and has approved it for publication.

## Conflict of Interest

The author declares that the research was conducted in the absence of any commercial or financial relationships that could be construed as a potential conflict of interest.

## References

[B1] Benítez-Burraco A. Murphy E. (2019). Why brain oscillations are improving our understanding of language. Front. Behav. Neurosci. 13:190. 10.3389/fnbeh.2019.00190 31551725 PMC6736581

[B2] Dayan P. Abbott L. F. (2001). Theoretical Neuroscience: Computational and Mathematical Modeling of Neural Systems. Cambridge, MA: MIT Press.

[B3] Ferguson K. A. Cardin J. A. (2020). Mechanisms underlying gain modulation in the cortex. Nat. Neurosci. 21, 80–92. 10.1038/s41583-019-0253-y 31911627 PMC7408409

[B4] Friston K. (2005). A theory of cortical responses. Philos. Trans. R. Soc. Lond. Ser. B Biol. Sci. 360, 815–836. 10.1098/rstb.2005.1622 15937014 PMC1569488

[B5] Friston K. (2018). Does predictive coding have a future? Nat. Neurosci. 21, 1019–1021. 30038278 10.1038/s41593-018-0200-7

[B6] Friston K. J. Rosch R. Parr T. Price C. Bowman H. (2017). Deep temporal models and active inference. Neurosci. Biobehav. Rev. 77, 388–402. 10.1016/j.neubiorev.2017.04.009 28416414 PMC5461873

[B7] Keitel A. Gross J. Kayser C. (2018). Perceptually relevant speech tracking in auditory and motor cortex reflects distinct linguistic features. PLoS Biol. 16:e2004473. 10.1371/journal.pbio.2004473 29529019 PMC5864086

[B8] Martin A. (2016). Language processing as cue integration: grounding the psychology of language in perception and neurophysiology. Front. Psychol. 7:120. 10.3389/fpsyg.2016.00120 26909051 PMC4754405

[B9] Martin A. E. (2020). A compositional neural architecture for language. J. Cogn. Neurosci. 38, 1–20. 10.1162/jocn_a_01552 32108553

[B10] Meyer L. Sun Y. Martin A. E. (2019). Synchronous, but not entrained: exogenous and endogenous cortical rhythms of speech and language processing. Lang. Cogn. Neurosci. 10.31234/osf.io/4s83k

[B11] Molinaro N. Lizarazu M. (2018). Delta(but not theta)-band cortical entrainment involves speech-specific processing. Eur. J. Neurosci. 48, 2642–2650. 10.1111/ejn.13811 29283465

[B12] Murphy E. (2015). The brain dynamics of linguistic computation. Front. Psychol. 6:1515. 10.3389/fpsyg.2015.01515 26528201 PMC4602109

[B13] Murphy E. (2016). A theta-gamma neural code for feature set composition with phase-entrained delta nestings. UCL Work. Pap. Linguist. 28, 1–23.

[B14] Murphy E. (2018). Interfaces (travelling oscillations) + recursion (delta-theta code) = language, in The Talking Species: Perspectives on the Evolutionary, Neuronal and Cultural Foundations of Language, eds Luef E. Manuela M. (Graz: Unipress Graz Verlag), 251–269.

[B15] Murphy E. (2020). The Oscillatory Nature of Language. Cambridge: Cambridge University Press.

[B16] Murphy E. Benítez-Burraco A. (2017). Language deficits in schizophrenia and autism as related oscillatory connectomopathies: an evolutionary account. Neurosci. Biobehav. Rev. 83, 742–764. 10.1016/j.neubiorev.2016.07.029 27475632

[B17] Poeppel D. Assaneo M. F. (2020). Speech rhythms and their neural foundations. Nat. Rev. Neurosci. 21, 322–334. 10.1038/s41583-020-0304-4 32376899

[B18] Salinas E. Abbott L. F. (2001). Coordinate transformations in the visual system: how to generate gain fields and what to compute with them. Prog. Brain Res. 130, 175–190. 10.1016/S0079-6123(01)30012-2 11480274

[B19] Woolnough O. Forseth K. J. Rollo P. S. Tandon N. (2019). Uncovering the functional anatomy of the human insula during speech. eLife 8:e53086. 10.7554/eLife.53086 31852580 PMC6941893

